# Signal-Analytics Modeling of Fluorescence Time-to-Detection for *E. coli* in Treated Wastewater: A Joint Censoring and Sensor Model Approach

**DOI:** 10.1021/acsomega.6c00397

**Published:** 2026-07-02

**Authors:** Charles Andre Haab, Jussiane Souza Silva, Thiago Alexandro Nascimento de Andrade, Maria Clara Bohn Silva, Geovana Mussato Mello, Darliana Mello Souza, Adriano Marques Jaime, Vandré Sonza Pinto, Caroline Pinto Rangel, Tiago Batista Tonon, Leandro Michels

**Affiliations:** † Department of Electrical Energy Processing, 28118 Federal University of Santa Maria, Avenida Roraima 1000, Santa Maria, Rio Grande do Sul 97105-900, Brazil; ‡ Department of Chemistry, 28118 Federal University of Santa Maria, Avenida Roraima 1000, Santa Maria, Rio Grande do Sul 97105-900, Brazil; § Department of Statistics, 28118 Federal University of Santa Maria, Avenida Roraima 1000, Santa Maria, Rio Grande do Sul 97105-900, Brazil; ∥ Department of Computer Science, 28118 Federal University of Santa Maria, Avenida Roraima 1000, Santa Maria, Rio Grande do Sul 97105-900, Brazil; ⊥ Department of Food Science and Technology, 28118 Federal University of Santa Maria, Avenida Roraima 1000, Santa Maria, Rio Grande do Sul 97105-900, Brazil

## Abstract

Coliform bacteria are widely used as indicators of fecal contamination, and pathogenic strains of *Escherichia coli* may pose substantial risks to human health. Virulent *E. coli* can cause clinical manifestations ranging from self-limiting gastroenteritis to severe systemic disease, including in otherwise healthy individuals. Accordingly, numerous studies have pursued strategies for the early detection and quantification of these pathogens in environmental and clinical settings. Motivated by a miniaturized fluorescence-based incubation, the Optical Bacterial Growth Sensor–Fluorescence Detector, which provides minute-resolved time-to-detection (TTD) readouts for *E. coli* in treated wastewater, we propose a new probabilistic model tailored to describe the distribution of TTD values. In practical terms, the model is intended to indicate when a fluorescence-signal increase should be treated as a detection event and how this information can support routine operation. Using 47 independent treated-wastewater assays, we extracted minute-resolved fluorescence-signal TTD values and fitted the proposed model by maximum likelihood. The proposed distribution provides a more faithful description of fluorescence-signal TTD behavior under treated-wastewater operating conditions, supports fit-for-purpose specification of the lower operational boundary of the assay counting window, and provides operational guidance for routine monitoring under the present sensing conditions. The fitted model indicates that most TTD values fall within an operational window of approximately 7–10 h, with a very low probability of detections beyond 12 h. We show that the model outperforms classical distributions commonly used to describe TTD data for *E. coli*. Key mathematical properties are derived to support the empirical results, and Monte Carlo evidence based on a parametric bootstrap corroborates the main findings. Overall, this framework expands the statistical toolkit for modeling sensor-based TTD phenomena associated with *E. coli* and supports routine monitoring.

## Graphical abstract

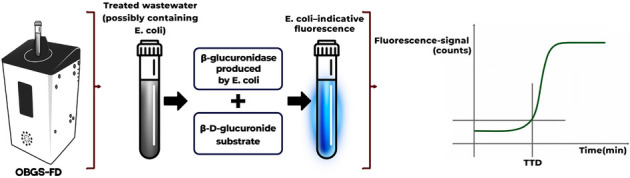

## 1 Introduction

*Escherichia coli* (*E. coli*) is a widely studied coliform bacterium, frequently used as an indicator of fecal contamination and associated public health risk. It remains a major concern because pathogenic variants can cause outcomes ranging from mild gastrointestinal illness to severe and potentially fatal diseases.^1–3^ In response, the specialized literature reports numerous initiatives aimed at detecting and quantifying *E. coli* in environmental and clinical settings, including approaches that monitor growth or metabolic curves generated by the microorganism.^4–6^ For this purpose, deterministic models from predictive microbiology, such as the Richards, Baranyi, and Roberts models, are frequently employed. However, purely deterministic formulations do not adequately capture the intrinsic variability of bacterial behavior and measurement processes, particularly when sensor signals are acquired from real wastewater samples.

Probability models provide a natural framework for quantifying uncertainty and variability in time-dependent phenomena, including time-to-event and detection-time data. In this broader context, probability distributions have been used as core modeling tools across diverse applied domains.^7–12^ Related developments have also appeared in environmental and sensor-signal applications,^13^ for example, they discuss distributions for peak flood discharges associated with atypical atmospheric, watershed, and fluvial processes, emphasizing heavy-tailed models and the Fréchet distribution. In turn, Fu et al.^14^ address fluorescence-signal fluctuations caused by suspended algal particles in natural waters through a continuous Poisson distribution model combined with statistical filtering, with results favoring this approach over Gaussian-based and classical frequency-domain filters.

Recent studies have also advanced rapid methods for *E. coli* detection in water-quality monitoring. Alonzo^15^ developed a microfluidic device and portable instrumentation for a phage-based assay that combines membrane filtration with selective enrichment using the genetically modified bacteriophage T7-NanoLuc-CBM, reducing analysis time to roughly one-third of that required by conventional methods while maintaining high sensitivity and a comparable limit of detection. Gómez^16^ evaluated a modified real-time reverse transcription polymerase chain reaction assay targeting the 16S rRNA gene for detecting *E. coli* in drinking water, reducing the time to result from 21–24 h to about 3 h in a multilaboratory European study. Finally, Cui et al.^17^ proposed a microwave biosensor for the direct detection of Shiga toxin-producing *E. coli* O157:H7 in aqueous samples, reporting a limit of detection of approximately 647 colony-forming units (CFU) mL^–1^ in deionized water, reducible to about 6.47 CFU mL^–1^ with a simple preconcentration step.

Against this background, the present study leverages this probabilistic perspective to address a sensor-analytics problem that arises in fluorescence-based monitoring of *E. coli*. More specifically, it proposes a practical method to translate high-frequency fluorescence readouts into actionable time-to-detection (TTD) thresholds under the present treated-wastewater sensing conditions. In practical terms, we ask when an increase in fluorescence should be treated as a true detection event, how variable this detection time is across experiments, and how this variability can be converted into routine monitoring rules for the instrument. Building on best-practice criteria reported in the biosensing and microbiological enumeration literature, we adopt an operational definition of TTD that accounts for baseline drift, measurement noise, and the early, subvisible stages of enzymatic signal emergence. The experimental motivation is a miniaturized, IoT-enabled incubation and fluorescence-detection platform that monitors enzymatic reactions between *E. coli* and the β-d-glucuronide substrate, yielding metabolic curves from treated wastewater samples.

The present study is restricted to treated wastewater and is not intended as a head-to-head assessment of overall sensor accuracy relative to alternative *E. coli* quantification methods. Instead, using 47 fluorescence-signal observations generated within the adopted microbiological workflow, we focused on the probabilistic modeling of TTD. Within this scope, sensor behavior at low *E. coli* levels is addressed through the distributional modeling of detection time rather than through fixed concentration-based analytical detection limits across different real wastewater samples.

Using multiple independent real wastewater samples, we introduce a previously unreported approach for probabilistic inference based on fluorescence-signal TTD in *E. coli* monitoring. Specifically, we propose the *Joint Censoring and Sensor Model Approach* (JCSMA), which integrates sensor-signal acquisition, operational TTD extraction, and probabilistic modeling of the resulting detection-time distribution. This framework supports a fit-for-purpose definition of decision rules and the characterization of the operational TTD window while remaining interpretable for routine monitoring and decision-making. Accordingly, the emphasis of the present paper is not only on model fit but also on when a run can be reported early, when it should continue, and how fluorescence-based detections can be interpreted under the sensing conditions considered here. Importantly, the objective is not to minimize TTD itself but to characterize its distribution so that detection events, early reporting opportunities, and run-duration decisions can be defined on a model-based basis.

The main sensor-oriented contributions of this work can, therefore, be articulated as an integrated sequence of methodological and operational advances. We adopt a clear operational definition of fluorescence-signal TTD thresholds, enabling consistent extraction of detection times under realistic sensing artifacts, including baseline drift, noise, and early subvisible signal emergence. We then introduce the JCSMA as an end-to-end framework connecting signal acquisition, TTD extraction, and probabilistic modeling within a single sensor analytics pipeline for *E. coli* monitoring. The approach is demonstrated by using independent real-world experiments with fluorescence-signal data acquired from treated wastewater samples, highlighting its applicability to matrices relevant to environmental surveillance. Within this framework, we propose a previously unexplored TTD distribution for fluorescence-signal TTD modeling in *E. coli* monitoring, constructed without adding parameters relative to the baseline model and, therefore, preserving parsimony while improving interpretability for sensor operation.

The resulting model provides an improved description of TTD data compared with classical parametric alternatives, including the gamma, log-logistic, Gompertz, and log-normal distributions, supporting fit-for-purpose characterization of the operational TTD window. Uncertainty and robustness are assessed through resampling-based and simulation-based validation, strengthening the reliability of the conclusions under realistic experimental variability. We also derive an analytical expression for $R=P\left(Y_{1}>Y_{2}\right)$ , which provides a concise reliability-based summary of comparative detection performance in the proposed framework. Finally, when coupled with the Optical Bacterial Growth Sensor–Fluorescence Detector (OBGS-FD),^18,19^ the proposed framework supports *E. coli* screening workflows by informing early reporting windows and run-duration decisions for routine monitoring under the present sensing conditions. This set of contributions motivates the organization of the remainder of the article.

## 2 Method

### 2.1 Experimental Setup

Samples are collected in duplicate at the wastewater treatment plant and immediately transported to the laboratory. Each sample is dispensed into autoclave-sterilized test tubes preloaded with a defined fluorogenic substrate. This reagent undergoes selective β-glucuronidase-catalyzed hydrolysis, yielding a fluorescent product that serves as an operational biochemical signature of *E. coli*—an approach that is extensively documented in the literature.^20–24^ The paired sample units were processed as follows: a 10 mL aliquot of treated effluent was introduced into the OBGS-FD equipment (Auftek, Brazil), in which the analytical response was derived from the time-dependent fluorescence signal (signal intensity, counts). A second aliquot of 100 mL was sent to an independent laboratory for comparative analysis using the Colilert/Quanti-Tray 2000 reference method, according to Standard Method 9223 B, with results expressed as the MPN (Most Probable Number) per 100 mL.^25^

The OBGS-FD equipment^18,19^ consists of a miniaturized laboratory setup that integrates IoT resources, electrical impedance measurements, and optical sensors for incubating treated wastewater samples that may harbor *E. coli*. Throughout the incubation period, the device records, in real time with 1 min resolution, the reaction progress associated with cumulative enzymatic substrate turnover. This biochemical readout is transduced by the system’s fluorescence sensors, which generate curves such as those illustrated in Figure 1. From these trajectories, features are extracted that enable estimation of the MPN of *E. coli*. For calibration purposes, both the lower and upper boundaries that define the beginning and the end of the counting window are essential. In the present study, these boundaries are understood in temporal-operational terms rather than as concentration-based analytical detection limits across real wastewater samples. However, the focus of this paper is the lower boundary, namely the TTD, which marks the onset of a sustained fluorescence signal increase. Under fixed assay and incubation conditions, this onset time is generally expected to vary with the initial bacterial burden, with earlier threshold crossing in higher-load samples and later threshold crossing in lower-load samples. Accurate statistical modeling of TTD is critical because even modest systematic errors in this onset time can propagate into the MPN calibration, leading, for instance, to biased quantification and an increased risk of false negatives at low bacterial loads, where the signal rise is subtle and more susceptible to baseline noise and drift.

**Figure 1:**
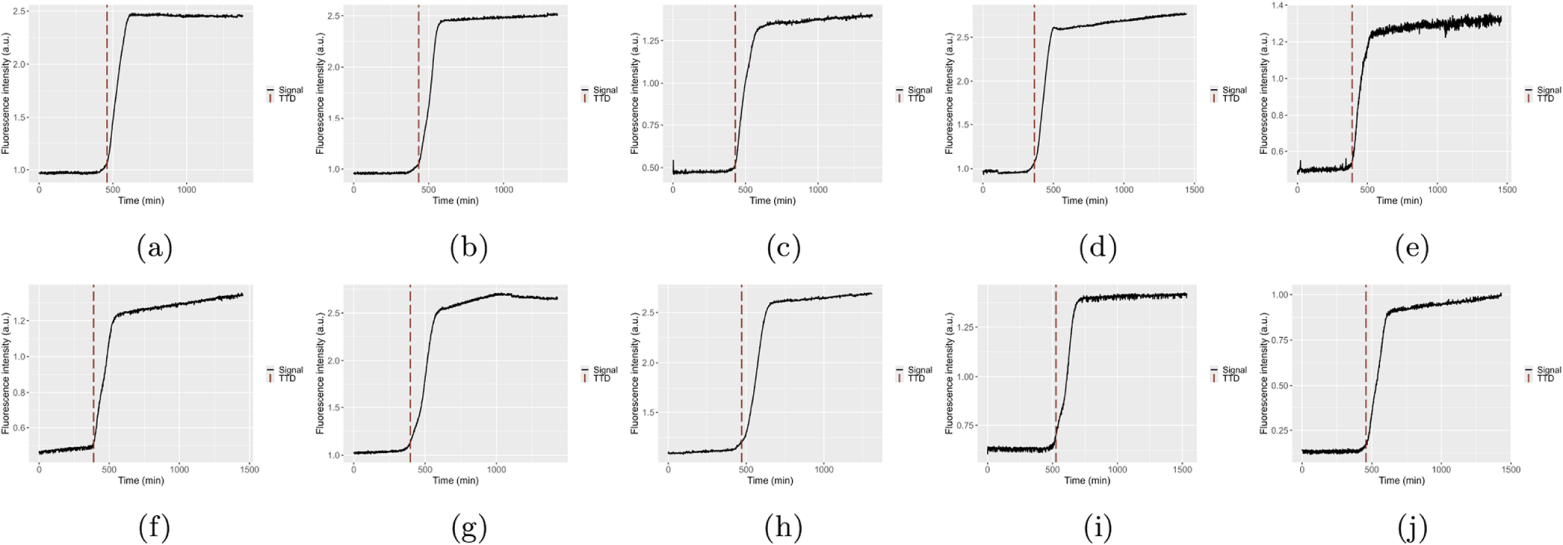
Representative fluorescence-signal time series and empirical TTD from real assays. Panels (a–j) show minute-resolved fluorescence-signal intensity (a.u.) recorded by the incubation sensing platform for independent experiments (wells). In each panel, the solid black curve is the measured fluorescence signal, which rises as the enzymatic reaction associated with *E. coli* progresses on a fluorogenic/chromogenic substrate. The vertical dashed line marks the empirically estimated TTD, defined as the onset time at which the signal becomes persistently associated with the metabolic reaction (see Definition 1 for the operational rule).

Figure 2 summarizes the complete sensing and analytics pipeline used in this study, spanning treated-wastewater sampling and incubation in a miniaturized fluorescence-based platform, minute-resolved signal acquisition, preprocessing, feature extraction, and computation of the time-to-detection (TTD) metric. In total, 47 treated-wastewater samples were analyzed, and a single TTD value was obtained for each assay from the corresponding fluorescence-signal time series recorded by the platform’s optical sensors. These 47 TTD measurements define the response variable modeled herein and provide the empirical basis for the sensor performance and operational TTD-window framework developed throughout the article.

**Figure 2:**
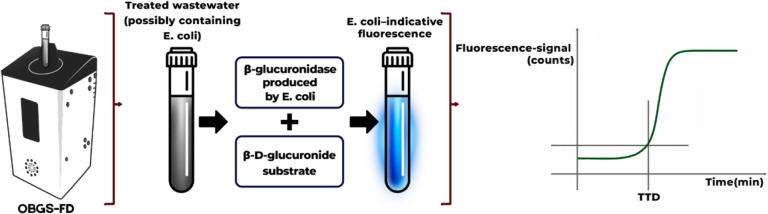
Experimental workflow and TTD concept. Treated wastewater samples are incubated with a fluorogenic β-d-glucuronide substrate. When *E. coli* is present, its β-glucuronidase hydrolyzes the substrate, generating an *E. coli*-indicative fluorescent product (left). The OBGS-FD records the fluorescence signal over time, producing a characteristic response curve from which the TTD is obtained as the onset of a sustained increase (right).

### 2.2 Time-to-Detection

The TTD is defined as the time at which the fluorescence signal becomes consistently attributable to the metabolic reaction detected by the sensor. Over an initial baseline window, we compute the median *B* and the median absolute deviation *MAD*_0_, and set the detection threshold to τ = max­{*B* + 5 MAD_0_, 1.1 *B*}. We then define *i*_0_ as the first index for which three consecutive points satisfy *s*_*i*_, *s*_*i*+1_, *s*_*i*+2_ > τ. If no such triplet exists, the TTD is set to NA. When *i*_0_ exists, the TTD is obtained by linear interpolation between $t_{i_{0}-1}$ and $t_{i_{0}}$ when the threshold crossing occurs within this interval, and set to $t_{i_{0}}$ otherwise. The operational rule adopted here to define TTD for fluorescence signal trajectories was guided by related detection practices reported in the literature and tailored to the present sensing setting.^26–35^ It is formalized in Definition 1 to make the empirical criterion used in this study explicit and reproducible.

**Definition 1** (Empirical TTD). Let $\{\left(t_{i},s_{i}\right){\}}_{i=1}^{n}$ be the fluorescence-signal series (time in minutes). On a baseline set $I_{0}$ (first 60 min, or if fewer than five observations, the first min­{*n*, max­(30, ⌈0.1 *n*⌉)} times), define $B=\mathrm{median}\left\{s_{i}:i\in I_{0}\right\},{\mathrm{MAD}}_{0}=\mathrm{median}\left\{|s_{i}-B|:i\in I_{0}\right\}$ and the threshold τ = max­{*B* + 5 MAD_0_, 1.1 *B*}. Let *i*_0_ = min­{*i*: *s*_*i*_, *s*_*i*+1_, *s*_*i*+2_ > τ}, if this set is nonempty (otherwise set TTD = NA). When *i*_0_ exists, the TTD is

1 $$\mathrm{TTD}=\left\{ \begin{array}{ll} t_{i_{0}-1}+\frac{\tau -s_{i_{0}-1}}{s_{i_{0}}-s_{i_{0}-1}}\left(t_{i_{0}}-t_{i_{0}-1}\right) & \mathrm{if}\;i_{0}>1,s_{i_{0}-1}\leq \tau <s_{i_{0}},\\ t_{i_{0}} & \mathrm{otherwise} \end{array}\right.$$

Table 1 reports the TTD values for the 47 independent experiments, and Table 2 summarizes the corresponding descriptive statistics.

**Table 1:** TTD Values (Minutes)

460.3667	433.5667	429.5500	387.1000	608.1500	378.6667	365.1000	390.5000
421.9667	395.7500	388.2500	407.3500	376.5200	374.0000	390.5000	395.6000
410.6500	395.0000	471.2333	434.1333	434.9000	320.5800	324.8500	613.7000
555.7500	527.0000	485.6333	533.3500	458.0000	458.6000	448.0500	540.5333
495.1000	541.8000	507.9000	503.0000	514.0333	570.1000	564.1000	544.7000
572.5667	665.5800	555.4000	595.1500	645.5000	197.6400	280.0889	–

**Table 2:** Descriptive Statistics for TTD (Minutes)

Statistic	Value
*n*	47
Mean	463.14
Median	458
Standard deviation	98.3
Skewness	–0.13
Kurtosis	2.87
Minimum	197.6
Maximum	665.6

The minimum and maximum TTD are 197.64 and 665.58 min, respectively, corresponding to detection times of roughly 3 to 11 h. The mean and median are very close, both near 8 h, and the standard deviation is moderate (about 1.5 h), indicating variability consistent with this central level. In terms of shape, the empirical distribution appears well-behaved: the sample shows near symmetry (skewness −0.13) and approximately mesokurtic behavior (kurtosis 2.87), with no pronounced departure from a Gaussian-like pattern.

### 2.3 The Proposed Distribution

In this subsection, we introduce the new probability model underlying the proposed framework for fluorescence-signal TTD data. To the best of our knowledge, this distribution has not been previously studied in the literature. Specifically, letting *Y* = 1/log­(1 + *X*), where *X* follows a Fréchet distribution with parameters λ > 0 and σ > 0, yields a two-parameter distribution on (0, ∞), which we denote by JCSMA­(λ, σ). Its cumulative distribution function (cdf) and probability density function (pdf) are given by

2 $$F_{Y}\left(y;\lambda ,\sigma \right)=1-\exp\left[-{(\frac{\sigma }{\phi \left(y\right)})}^{\lambda }\right],\;y>0$$

and

3 $$f_{Y}\left(y;\lambda ,\sigma \right)=\lambda \sigma^{\lambda }\frac{\phi \left(y\right)+1}{y^{2}}\phi {\left(y\right)}^{-(\lambda +1)}\exp\left[-{(\frac{\sigma }{\phi \left(y\right)})}^{\lambda }\right],\;y>0$$

respectively, where ϕ­(*y*) = *e* ^1/*y*^ – 1.

These two expressions constitute the main probabilistic components proposed in this work for TTD modeling, as they describe both the cumulative behavior of detection times and their distribution over the positive support. In this way, the model provides a direct probabilistic description of how fluorescence-signal TTD values are expected to behave, including their concentration, asymmetry, and tail behavior. A formal statement of this construction, including its derivation from the Fréchet distribution and the corresponding proof, is provided in the Supporting Information.

Throughout the paper, we write *Y* ∼ JCSMA­(λ, σ) when *Y* has cdf eqs 2 and pdf [Disp-formula eq3]. Representative density plots for selected parameter values are shown in Figure 3, illustrating that the model can accommodate a range of shapes, including approximately symmetric and asymmetric forms, as well as lighter- and heavier-tailed behavior. This flexibility is particularly useful for modeling fluorescence-signal TTD data.

**Figure 3:**
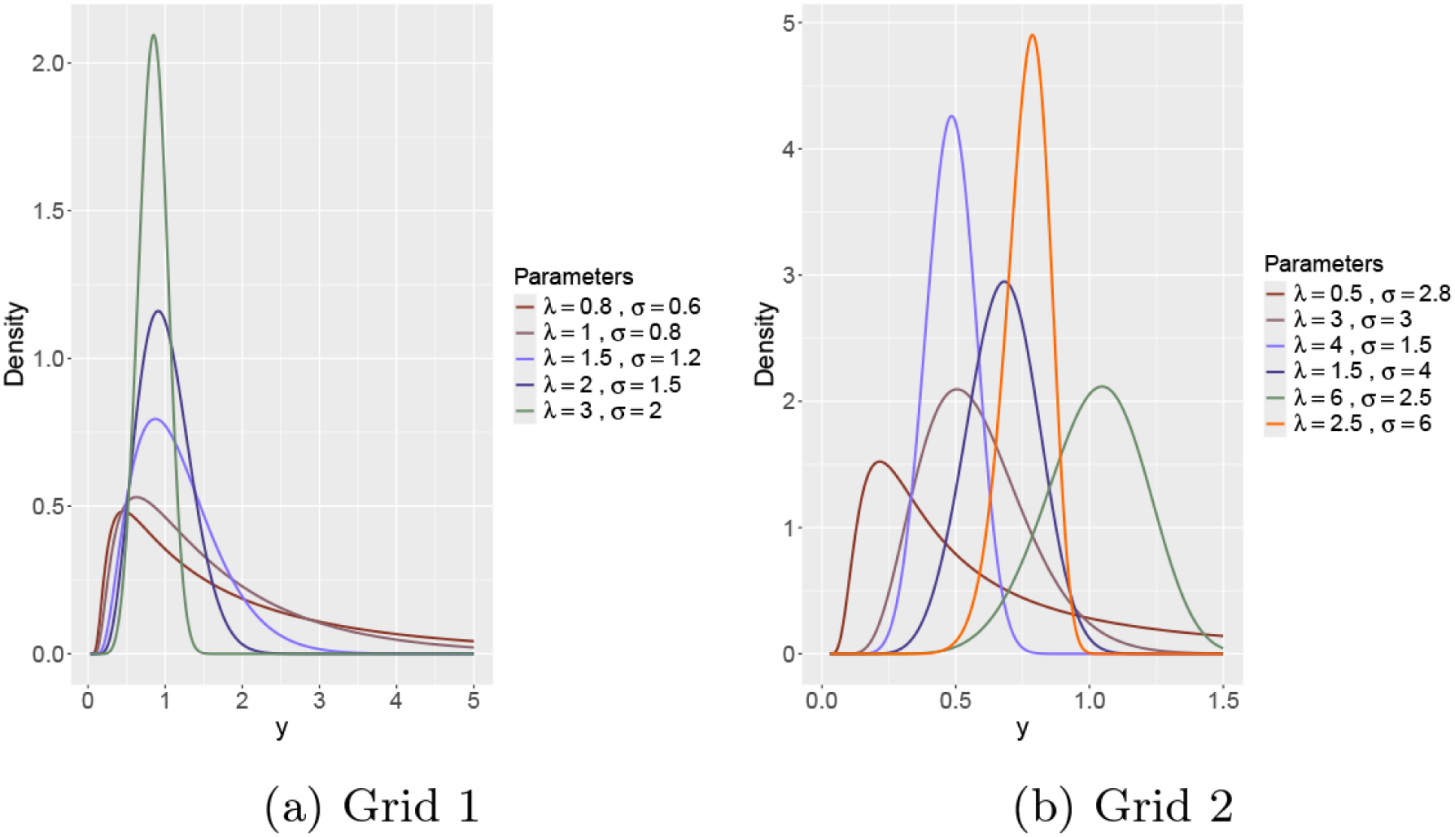
Estimated densities of the new JCSMA model for different parameter pairs (λ, σ): (a) Grid 1. (b) Grid 2.

For the random variable *Y* defined above, the survival function, the cumulative hazard function, and the hazard rate take the following forms:

4 $${\mathrm{F̅}}_{Y}\left(y;\lambda ,\sigma \right)=\exp\left[-{(\frac{\sigma }{\phi \left(y\right)})}^{\lambda }\right]$$

5 $$\Lambda_{Y}\left(y;\lambda ,\sigma \right)={(\frac{\sigma }{\phi \left(y\right)})}^{\lambda }$$

and,

6 $$h_{Y}\left(y;\lambda ,\sigma \right)=\lambda \sigma^{\lambda }\frac{\phi \left(y\right)+1}{y^{2}}\phi {\left(y\right)}^{-(\lambda +1)}$$

As with the cdf and pdf, eqs 4–6 provide a unique characterization of the random variable *Y*. The practical use of these functions is highlighted in Section 3. To facilitate exploration of the flexibility of the proposed model, we provide an interactive web application developed in Shiny^36^ in which the density and hazard functions of the JCSMA model can be evaluated. The application is available at https://jcsma.shinyapps.io/JCSMA/.

For many practical applications, it is essential to define the quantile function (qf), understood as the inverse of the cdf. Through this function, important characterizations can be obtained, such as moments, measures of skewness,^37^ and kurtosis,^38^ as well as the generation of realizations of the random variable *Y* via the inversion method, among other uses. The qf of the JCSMA model is used to construct the theoretical QQ-plot displayed in Figure 4 in Section 3, and it admits the closed-form expression given by

7 $$Q_{Y}\left(u;\lambda ,\sigma \right)=\{\log\left\{1+\frac{\sigma }{{[-\log(1-u)]}^{1/\lambda }}\right\}{\}}^{-1},,0<u<1$$

**Figure 4:**
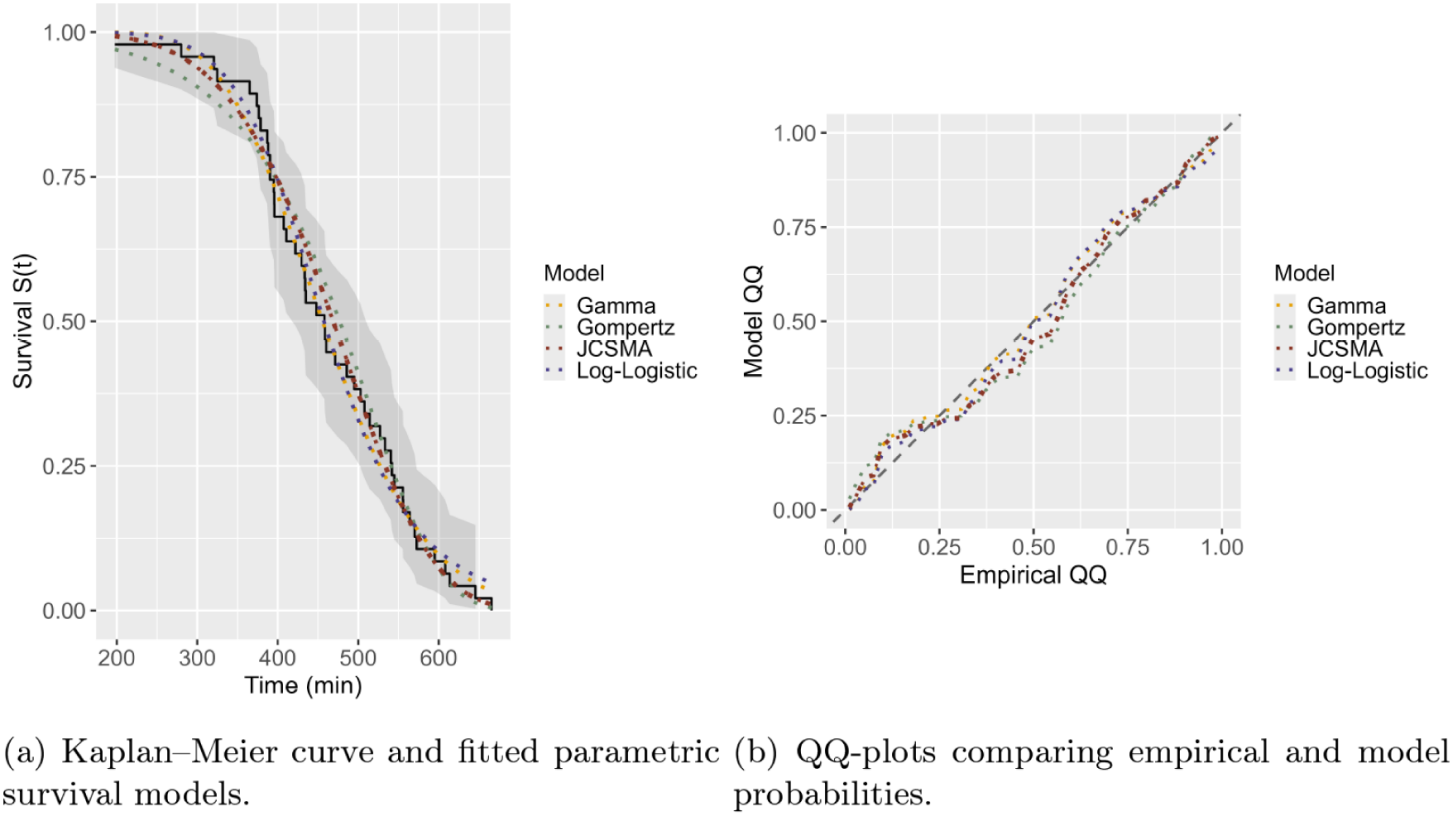
Graphical assessment of the parametric models fitted to the TTD data. (a) Kaplan–Meier curve and fitted parametric survival models. (b) QQ-plots comparing empirical and model probabilities.

In many applied sensor settings, it is useful to quantify probabilities of the form $R=P\left(Y_{1}>Y_{2}\right)$ . This quantity is referred to in the specialized literature as the reliability measure or stress–strength parameter. In what follows, we consider *Y*_1_ ∼ JCSMA­(λ_1_, σ_1_) and *Y*_2_ ∼ JCSMA­(λ_2_, σ_2_) as independent random variables on (0, ∞), with cdf and pdf given, respectively, by eqs 2 and q [Disp-formula eq3]. Let *f*_1_(*y*; λ_1_, σ_1_) denote the probability density function of *Y*_1_ and *F*_2_(*y*; λ_2_, σ_2_) the cumulative distribution function of *Y*_2_. The reliability can then be written as

8 $$R=\int_{0}^{\infty }f_{1}\left(y;\lambda_{1},\sigma_{1}\right)F_{2}\left(y;\lambda_{2},\sigma_{2}\right)dy$$

From a sensor analytics standpoint, $R=P\left(Y_{1}>Y_{2}\right)$ provides a compact, operationally meaningful metric for comparing TTD behavior across two sensing conditions, such as different MPN levels, real wastewater samples, incubation protocols, or instrument settings. In the present work, TTD is used as the primary response variable to define detection events, characterize the operational TTD window, and support early reporting and run-duration decisions under the adopted sensing protocol. Because *Y* is a monotonic index derived from the TTD readout, smaller values of *Y* indicate an earlier detection under the same acquisition and processing rules. Accordingly, R represents the probability that a randomly selected experiment from condition 1 yields a larger Y than a randomly selected experiment from condition 2. Thus, R summarizes relative detection timing across conditions, rather than overall analytical performance,and is used here to support fit-for-purpose calibration and monitoring decisions.

In general, the stress–strength reliability $R=P\left(Y_{1}>Y_{2}\right)$ does not admit a closed-form expression over the full parameter space of the JCSMA model and can be evaluated numerically. However, a simple analytical expression is available in the important special case where the two independent JCSMA variables share the same shape parameter. Specifically, if *Y*_1_ ∼ JCSMA­(λ, σ_1_) and *Y*_2_ ∼ JCSMA­(λ, σ_2_), with σ_1_, σ_2_, λ > 0, then

9 $$R=P\left(Y_{1}>Y_{2}\right)=\frac{\sigma_{2}^{\lambda }}{\sigma_{1}^{\lambda }+\sigma_{2}^{\lambda }}$$

This result provides a convenient closed-form characterization of the reliability measure in the equal shape setting, which is useful for interpretation and computation. A formal statement and derivation of this expression are provided in the Supporting Information.

Table 3 reports reliability values for the JCSMA stress–strength model for selected parameter combinations, comparing the results obtained from eqs 8 and 9. Two main findings emerge: (i) the reliability estimates obtained via numerical integration and via the closed-form expression agree to at least six decimal places, and (ii) when λ_1_ = λ_2_ and σ_1_ = σ_2_, the reliability equals 0.5, as expected, since in this case *Y*_1_ and *Y*_2_ follow identical distributions.

**Table 3:** Numerical Reliability for the JCSMA Stress–Strength Model

σ_1_	λ_1_	σ_2_	λ_2_	R̂ (numeric)	Closed-form	Abs. error
0.5	0.5	0.5	0.5	0.500000	0.500000	6.95e-13
1.0	1.0	1.0	1.0	0.500000	0.500000	6.95e-13
1.5	2.0	1.5	2.0	0.500000	0.500000	6.95e-13
2.0	0.75	2.0	0.75	0.500000	0.500000	6.95e-13
1.0	1.0	2.0	1.0	0.666667	0.666667	3.05e-12
2.0	1.0	1.0	1.0	0.333333	0.333333	3.65e-12
0.8	1.5	1.2	1.5	0.647530	0.647530	5.31e-12
1.2	1.5	0.8	1.5	0.352470	0.352470	3.19e-12

The specialized literature is replete with estimation methods that enable robust inference on the parameter vector associated with a statistical model. Nevertheless, the maximum likelihood method^39,40^ remains the most widely used in practical applications. Let *y*_1_, ..., *y*_*n*_ denote an observed sample of size *n* from the JCSMA distribution. The log-likelihood function for the parameter vector **ϒ** = (λ, σ)^T^, denoted l(Υ) , is given by

10 $$l\left(\Upsilon \right)=n\left(\log\lambda +\lambda \log\sigma \right)+\overset{n}{\underset{i=1}{\sum }}\left[\log\left(\phi \left(y_{i}\right)+1\right)-2\log y_{i}-\left(\lambda +1\right)\log\phi \left(y_{i}\right)\right]-\overset{n}{\underset{i=1}{\sum }}{\left(\frac{\sigma }{\phi \left(y_{i}\right)}\right)}^{\lambda }$$

The maximum likelihood estimators (MLEs) ***Υ̂*** = (*λ̂*, *σ̂*)^T^ for the parameters of the JCSMA model are obtained as the solution to the system of likelihood equations $\frac{\partial l\left(\Upsilon \right)}{\partial \lambda }=0$ and $\frac{\partial l\left(\Upsilon \right)}{\partial \sigma }=0$ . Unfortunately, the MLEs do not admit closed-form analytical expressions. Nevertheless, they can be computed conveniently using numerical optimization routines available in standard R scripts.^41^

## 3 Results and Discussion

In this subsection, we present the results of applying the proposed model to the TTD data, summarized in Table 1. The MLEs were obtained by implementing eq 10 in the R programming language.^41^ The maximization procedure was carried out using the function flexsurv::flexsurvreg­(), which fits parametric survival models with right-censored data by maximizing the corresponding log-likelihood. For the new JCSMA model, the parameter estimates with their 95% confidence intervals (CIs) are *σ̂* = 0.0020 (0.0019, 0.0021) and *λ̂* = 5.3607 (4.2974, 6.6870). The estimates and CIs for the competing models are available upon request. To assess the goodness-of-fit of the proposed model, we considered, as competitors, classical two-parameter models from the literature, namely the gamma, log-logistic and Gompertz distributions, which are widely used for modeling growth curves in practical applications. Figure 4 displays the empirical Kaplan–Meier survival curve together with the fitted parametric survival curves for each model, as well as the corresponding QQ-plots.

Visually, all of the models appear to provide an adequate description of the data. However, a quantitative comparison requires the use of formal model-selection criteria. In this study, we employed the Akaike Information Criterion (AIC) and the Bayesian Information Criterion (BIC), for which, in general, smaller values indicate a preferable model. Table 4 reports the AIC and BIC values for all models under consideration, and based on these results, the proposed model can be selected as the most appropriate one to describe the probabilistic behavior of the TTD data. From an operational standpoint, however, the key issue is not only which model fits the data better but also what monitoring windows and reporting cutoffs the fitted distribution supports under the present sensing conditions.

**Table 4:** Information Criteria for Fitted Parametric TTD Models

Distribution	logLik	AIC	BIC
JCSMA	–**281.8801**	**567.7603**	**571.4606**
Gamma	–283.1953	570.3907	574.0910
Log–Logistic	–283.5151	571.0302	574.7305
Gompertz	–283.7813	571.5626	575.2629
Log-Normal	–284.5991	573.1983	576.8986
Inverse Gaussian	–284.8465	573.6929	577.3932
Gumbel	–286.6122	577.2244	580.9247
Fréchet	–296.1161	596.2322	599.9325
Rayleigh	–306.0813	614.1625	616.0127
Exponential	–335.4873	672.9747	674.8248
Pareto	–335.4913	674.9825	678.6828

Once the adequacy of the JCSMA model has been established, it can be directly leveraged in practical sensing scenarios to define fit-for-purpose operational windows and to support instrument calibration, quality control checks, and routine decision-making in real wastewater samples. In particular, by plugging the MLEs into eqs 2 and 4, one can compute probabilities of the form P(Y<y) and P(Y>y) , respectively, thereby translating the fitted model into interpretable statements about the distribution of TTD for the fluorescence-based assay under the current incubation and readout conditions. These probability statements are especially useful for formalizing reporting rules (e.g., early reporting windows) and for defining stopping times under the present sensing conditions, thereby characterizing the operational TTD window rather than minimizing TTD itself.

Using these expressions, we obtain, for instance, P(Y>720)=0.0010 , indicating that it is highly implausible for the TTD to exceed 12 h under the present sensing and incubation conditions. Conversely, it is very likely that the TTD associated with a given assay is shorter than 9 h, since P(Y<540)=0.7719 . From an operational standpoint, such tail probability and cumulative probability calculations provide actionable guidance for setting monitoring windows, specifying reporting cutoffs, and anticipating TTD in routine workflows. More broadly, they complement conventional performance descriptors by quantifying uncertainty in the timing of a detectable response, which is often the central metric in field-deployable and near-real-time sensing applications.

To complement these pointwise probability calculations, Table 5 reports probability calculations for TTD ranges that are informative for practical interpretation and method operation. For instance, P(400<Y<600)=0.6701 indicates an approximate 67% chance that, for a given sample, the TTD lies between about 7 and 10 h, which can be used to delineate an operational window for early reporting and to guide calibration and routine monitoring in real wastewater samples. Likewise, the lower probability assigned to the 600–720 min interval helps quantify the expected frequency of late-detection events, supporting the specification of maximum run times and reporting cutoffs with a transparent, model-based rationale.

**Table 5:** Probabilities for Operational TTD Intervals under the JCSMA Model

*a* (min)	*b* (min)	P(a<Y<b)
300	400	0.1946
360	480	0.3897
400	500	0.3683
400	600	0.6701
500	600	0.3019
600	720	0.0732

Taken together, these pointwise and interval-based results indicate that, under the present treated wastewater sensing conditions, the empirical TTD values ranged from 197.64 to 665.58 min (approximately 3.3 to 11.1 h), and the fitted JCSMA model assigns only a 0.0010 probability to TTD values exceeding 720 min. Accordingly, the calibration-relevant output of the present framework is temporal-operational: it delineates the practical TTD window within which fluorescence-based detections are expected and within which reporting and run-duration decisions can be made. It should not be interpreted as a concentration-based analytical calibration range in MPN or CFU units, since the present study was not designed as a concentration-stratified validation of overall sensor accuracy across methods or matrices.

## 4 Monte Carlo Parametric Bootstrap TTD

In this section, we further investigate the applied results obtained in Section 3. Assuming that the JCSMA model is the correct model and replacing the parameters with their MLEs, we use the quantile function defined in eq 7 and 5000 Monte Carlo (see refs 42–44 ) replications to generate TTD samples via parametric bootstrap (see refs 45–47 ) of size *n* = 47 (matching the original sample size). For each replication, the parameters are treated as unknown, the model is refitted, and AIC is computed. At the end of this process, the empirical distributions of the sample mean, standard deviation, skewness, kurtosis, and AIC values are obtained and plotted. The resulting distributions, shown in Figure 5, support the adequacy of the proposed model for the data under study, since the simulated empirical distributions are compatible with the reference descriptive statistics in Table 2 as well as with the empirical AIC reported in Table 4. A pseudocode algorithm presented in the Supporting Information material ensures the reproducibility of this experiment.

**Figure 5:**
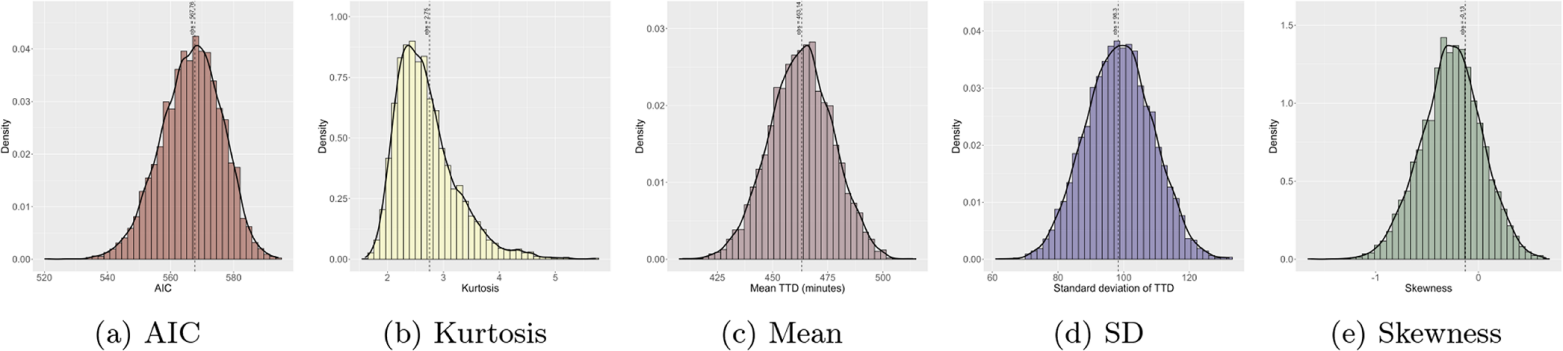
Monte Carlo distributions of key summary statistics under the fitted JCSMA model. Histograms show the parametric bootstrap distributions of (a) AIC, (b) sample kurtosis, (c) sample mean, (d) sample standard deviation, and (e) sample skewness of the TTD data. Solid curves denote kernel density estimates, and vertical dashed lines mark the values observed in the original TTD sample.

## 5 Conclusions

*E. coli* is a pathogenic member of the coliform group, and its variants can affect human health across a wide range of severities. For this reason, the specialized literature frequently proposes alternative methods for the detection and quantification of this bacterium, including fluorescence-based sensing strategies that generate time-to-detection (TTD) information on real wastewater samples. Nonetheless, the development of statistical models that adequately capture TTD behavior and translate it into fit-for-purpose calibration guidance for sensor operation remains relatively underexplored. In this study, we advance the current state of the art by proposing a new probability distribution to model TTD values derived from growth curves associated with cumulative enzymatic processes of *E. coli* in treated wastewater samples. Real data are obtained from miniaturized fluorescence-based incubation platforms that generate minute-by-minute fluorescence readouts and corresponding TTD values for *E. coli*. In practical terms, the proposed model helps determine when fluorescence signal increases can be treated as detection events and how these times can be interpreted for routine instrument operation under the present sensing conditions. Several mathematical properties derived for the new model support accurate inference and, consequently, more robust, operationally interpretable decision making. In contrast to classical distributions, standard goodness-of-fit measures favor the model proposed in this study. Monte Carlo simulations based on a parametric bootstrap scheme are consistent with and reinforce the empirical results.

The efforts of this study, which focus on characterizing the lower operational boundary of the fluorescence-signal TTD window rather than fixed-concentration-based analytical detection limits across real wastewater samples, are complemented by an ongoing investigation aimed at modeling the upper boundary of the same operational window within the same fluorescence-signal TTD framework. Although these two contributions are conceptually aligned and mutually reinforce each other, they are methodologically independent. As such, applied researchers may benefit from the present findings in isolation, from the forthcoming results on the upper operational boundary, or from the combined use of both models, depending on the specific requirements of their calibration, monitoring, and decision-making protocols.

## Supplementary Material



## Notes

The formal derivation of the JCSMA distribution from the Fréchet model, including the cumulative distribution function and probability density function, as well as the closed-form reliability expression for the equal-shape case. It also includes the pseudoalgorithm used for the Monte Carlo parametric bootstrap procedure adopted to assess the adequacy and reproducibility of the proposed TTD modeling framework (PDF)
