# Supplementary material for: Signal-Analytics Modeling of Fluorescence Time-to-Detection for E. coli in Treated Wastewater: A Joint Censoring and Sensor Model Approach
Source: ACS Omega. 2026 Jul 2;11(28):41534–42. doi: 10.1021/acsomega.6c00397 (PMC13393204; doi:10.1021/acsomega.6c00397)
Supplement: Supplementary file 1 [file ao6c00397_si_001.pdf]

Supporting Information

Signal-analytics modeling of fluorescence time-to-detection for *E. coli* in treated wastewater: a Joint Censoring & Sensor Model Approach

Charles Andre Haab[1], Jussiane Souza Silva*[2], Thiago A. N. De Andrade[3], Maria Clara Bohn Silva[4], Geovana Mussato Mello[2], Darliana Mello Souza[2], Adriano Marques Jaime[1], Vandré Sonza Pinto[5], Caroline Pinto Rangel[5], Tiago Batista Tonon[4], and Leandro Michels[1]

[1]Department of Electrical Energy Processing, Federal University of Santa Maria, Avenida Roraima 1000, 97105-900, Santa Maria, RS, Brazil
[2]Department of Chemistry, Federal University of Santa Maria, Avenida Roraima 1000, 97105-900, Santa Maria, RS, Brazil
[3]Department of Statistics, Federal University of Santa Maria, Avenida Roraima 1000, 97105-900, Santa Maria, RS, Brazil
[4]Department of Computer Science, Federal University of Santa Maria, Avenida Roraima 1000, 97105-900, Santa Maria, RS, Brazil
[5]Department of Food Science and Technology, Federal University of Santa Maria, Avenida Roraima 1000, 97105-900, Santa Maria, RS, Brazil

Contents of the Supporting Information

A Pseudo-codes for the Monte Carlo parametric bootstrap TTD procedures **3**

B Supplementary theoretical results for the JCSMA model **4**

A Pseudo-codes for the Monte Carlo parametric bootstrap TTD procedures

Algorithm 1 Monte Carlo procedure to obtain bootstrap distributions of AIC and summary statistics under the JCSMA model

Given the observed TTD sample $\{y_1, \ldots, y_n\}$
Fit the JCSMA model to $\{y_i\}$ by maximum likelihood and obtain the MLEs $(\hat{\lambda}, \hat{\sigma})$ and the observed AIC, AIC_{obs}
Compute the observed sample mean, standard deviation, skewness, and kurtosis
Choose the number of Monte Carlo replications B (e.g., $B = 5000$) and set a random seed
for $b = 1, \ldots, B$ **do**
    Generate a bootstrap sample $\{y_1^{(b)}, \ldots, y_n^{(b)}\}$ from the JCSMA model with parameters $(\hat{\lambda}, \hat{\sigma})$ using the inverse CDF
    Refit the JCSMA model to $\{y_i^{(b)}\}$ by maximum likelihood and store the bootstrap AIC value $\text{AIC}^{(b)}$
    Compute and store the sample mean, standard deviation, skewness, and kurtosis of $\{y_i^{(b)}\}$, denoted $\text{mean}^{(b)}$, $\text{sd}^{(b)}$, $\text{skew}^{(b)}$, and $\text{kurt}^{(b)}$
end for
for each statistic $S \in \{\text{AIC}, \text{mean}, \text{sd}, \text{skewness}, \text{kurtosis}\}$ **do**
    Construct the Monte Carlo distribution of S from $\{S^{(1)}, \ldots, S^{(B)}\}$
    Plot the histogram and a kernel density estimate of this distribution
    Superimpose a vertical dashed line at the corresponding observed value S_{obs} and annotate its numerical value
    Export the resulting panel as an image file to be included in the manuscript
end for

B Supplementary theoretical results for the JCSMA model

Theorem B.1 (New probabilistic model)**.** *Let X be Fréchet(λ, σ) on $(0, \infty)$ with cumulative distribution function (cdf)*

$$F_X(x; \lambda, \sigma) = \exp\Big[-\Big(\tfrac{\sigma}{x}\Big)^{\lambda} \Big] \mathbf{1}_{(0,\infty)}(x), \qquad \lambda > 0,\ \sigma > 0.$$

Define $Y = 1/\log(1 + X)$ and $\phi : (0, \infty) \to (0, \infty)$ by $\phi(y) = e^{1/y} - 1$. Then Y has support $(0, \infty)$ and is absolutely continuous with cdf

$$F_Y(y; \lambda, \sigma) = \left\{ 1 - \exp\Big[-\Big(\tfrac{\sigma}{\phi(y)}\Big)^{\lambda} \Big] \right\} \mathbf{1}_{(0,\infty)}(y), \tag{1}$$

and probability density function (pdf)

$$f_Y(y; \lambda, \sigma) = \left\{ \lambda\, \sigma^{\lambda}\, \frac{\phi(y) + 1}{y^2}\, \phi(y)^{-(\lambda+1)} \exp\Big[-\Big(\tfrac{\sigma}{\phi(y)}\Big)^{\lambda} \Big] \right\} \mathbf{1}_{(0,\infty)}(y). \tag{2}$$

Consequently, the pair formed by $F_Y(y; \lambda, \sigma)$ and $f_Y(y; \lambda, \sigma)$ defines and uniquely characterizes a new two-parameter, absolutely continuous probability distribution on $((0, \infty), \mathcal{B}((0, \infty)))$.

Proof. Because $y = 1/\log(1 + x)$ is strictly decreasing on $(0, \infty)$ and admits the inverse $x = \phi(y) = e^{1/y} - 1$, (1) follows from $F_Y(y) = \Pr(X \geq \phi(y))$. The density in (2) is then obtained by the change-of-variables formula. □

Theorem B.2 (Equal-shape reliability)**.** *Let $Y_1 \sim$ JCSMA(λ, σ_1) and $Y_2 \sim$ JCSMA(λ, σ_2) be independent random variables on $(0, \infty)$, with $\sigma_1, \sigma_2, \lambda > 0$, and define the stress–strength reliability $\mathcal{R} = \mathbb{P}(Y_1 > Y_2)$. Then*

$$\mathcal{R} = \frac{\sigma_2^{\lambda}}{\sigma_1^{\lambda} + \sigma_2^{\lambda}}. \tag{3}$$

Proof. The proof follows by direct integration and is omitted for brevity. □
